# Why is it easier to predict the epidemic curve than to reconstruct the underlying contact network?

**DOI:** 10.1007/s00285-026-02401-6

**Published:** 2026-05-02

**Authors:** Dániel Keliger, Illés Horváth

**Affiliations:** 1 Department of Stochastics, Budapest University of Technology and Economics, Budapest, Hungary; 2 Alfréd Rényi Institute of Mathematics, Budapest, Hungary; 3 Department of Networked Systems and Services, Budapest University of Technology and Economics, Budapest, Hungary; 4 HUN-REN-BME Information Systems Research Group, Budapest, Hungary

## Abstract

We study the deterministic Susceptible-Infected-Susceptible (SIS) epidemic model on weighted graphs. van Mieghem et al. have shown that it is possible to learn an estimated network from a finite time sample of the trajectories of the dynamics that in turn can give an accurate prediction beyond the sample time range, even though the estimated network might be qualitatively far from the ground truth. We give a mathematically rigorous derivation for this phenomenon, notably that for large networks, prediction of the epidemic curves is robust, while reconstructing the underlying network is ill-conditioned. Furthermore, we also provide an explicit formula for the underlying network when reconstruction is possible. At the heart of the explanation, we rely on Szemerédi’s weak regularity lemma.

## Introduction

One of the main approaches of epidemic modeling is compartment-based system of differential equations where the compartments correspond to geographic locations or age groups in different stages of the infection (Gozzi et al. 2021). Besides the initial conditions, infection and cure rates, arguably, the most challenging parameter to estimate is the underlying contact network on which the spreading phenomenon occurs. Direct methods usually rely on making modeling assumptions or using proxy networks based on social media networks, GPS and mobile data, commuting patterns or questioners (Islam et al. 2014; Ódor et al. 2021), methods which can be potentially intrusive in terms of privacy or give an unfaithful representation.

An alternative paradigm treats network estimation as a learning problem where we first use observations up to some time $T_0>0$ to find the parameters that are most consistent with the epidemic curves thus far, then using said parameters to extrapolate the curve into a future time $T>T_0$ (Prasse and Mieghem 2022). Surprisingly, while this method yields an estimated network capable of making accurate predictions for a reasonably long time scale, the realized estimate might radically differ from the ground truth alluding to the fact that only partial information of the underlying network is essential in the evolution of the epidemic. In summary, they show that while the *reconstruction* of the network is hard, *predicting* the epidemic curves is robust.

In Prasse and Mieghem (2022) the phenomena was demonstrated numerically along with theoretical arguments. In Beaufort et al. (2022) the authors study a slightly different setting where instead of commuting (individuals returning to their home vertex at a given rate (Gozzi et al. 2021)), individuals perform random walks according to a diffusion matrix. They rigorously prove that almost every diffusion matrix from almost every initial condition can be reconstructed from the trajectories and numerically show that said reconstruction can be challenging. Reconstruction of the background network graphon based on the distribution of branching processes was examined in Hladký et al. (2024). Murphy et al. (2024) provides a different setup with a stochastic process on a random graph, with an entropy inequality implying a tradeoff between prediction and reconstruction. Intuitively, if the evolution of the epidemic is sensitive to the details of the network, then one can extract a rich picture from the trajectories, while prediction becomes hard as even slight perturbations of the network results in a drastic change of dynamic. On the other hand, if many realizations of the random graph give similar trajectories, extrapolation becomes more feasible.

In the present paper, we follow the setting of Prasse and Mieghem (2022) for the Susceptible-Infected-Susceptible (SIS) model and give a proper mathematical setup and rigorous proof that shows that the prediction problem is robust, while the reconstruction problem is ill-conditioned for large networks. To the best of our knowledge, these results are the first in this endeavor. Furthermore, for some cases we also give an explicit formula to reconstruct the underlying network, using only observable quantities (quantities that can be observed or measured directly).

We believe the concepts and methods are robust for generalization to other kinds of models; an important notion is the observable quantities, and irregularities of the evolution also potentially play a role. These could be examined on a case-by-case basis; in the present paper, we do not pursue a general framework.

The rest of the paper is structured as follows: In Section 2 we give an introduction of the SIS model and rigorously define the reconstruction and prediction problems. In Section 3 we state the main results. Section 4 introduces concepts and notation regarding graphons (Lovász 2012) which are crucial for the proofs but non-essential in terms of stating the problems and the corresponding theorems. Section 5 contains the proofs for the main results.

## Setup

In this section we give a precise formulation of the model and the problems of interest.

### SIS model definition

We study the deterministic SIS metapopulation model. For simplicity of presentation, we choose to focus on SIS model, but most of the results can be extended to a wider class of epidemic models including the SIR or SEIR processes with appropriate modifications (e.g. to the set of observable quantities).

Assume there are *n* communities represented by the vertex set $V= \{1, \dots , n \}$. The graph is equipped with both symmetric edge weights $w_{ij}=w_{ji}\ge 0$ forming the matrix $W:= \left( w_{ij}\right) _{i,j \in V}$. Symmetry is usually assumed for similar epidemic models, but is actually not necessary for most of the calculations.

Positive vertex weights $\pi _{i} \ge 0$ are also given with normalization

$$\begin{aligned} \sum _{i=1}^{n} \pi _i =1. \end{aligned}$$

The vector $\pi := \left( \pi _{i} \right) _{i \in V}$ is referred to as the distribution of the population between communities. We also use the matrix notation $\Pi := \operatorname {diag} \left( \pi \right)$.

$w_{ij}$ represents the strength of interaction between communities $i,j \in V$. It can also be proportional to the edge density if the weighted graph corresponds to a coarse grained version of a simple graph. Global constants like the infection rate $\beta$ can also be absorbed in $w_{ij}$.

$\gamma _i \ge 0$ denotes the curing rate of community $i \in V$; the corresponding vector notation is $\gamma := \left( \gamma _i \right) _{i \in V}.$ Inhomogeneity of the curing rate might be an important modeling aspect when communities correspond to age groups or other biological characteristics of the population that can affect the curing rate.

The special case $\gamma =0$ is referred to as the SI model.

In the SIS model, individuals can occupy one of two states: susceptible and infected. Let $z_i(t)$ stand for the ratio of infected individuals at community $i \in V$ at time $t \ge 0.$

Due to the cure rate $\gamma _i$ the ratio $z_i(t)$ is decreased at rate $\gamma _{i} z_{i}(t)$. $z_i(t)$ is increased when a susceptible individual from community *i* makes an interaction with an infected individual from some community *j*. Assuming that the communities are well-mixed, the probability that the vertex from community *i* is susceptible and the vertex from community *j* is infected is $(1-z_i(t))z_{j}(t)$. Finally, $w_{ij} \pi _{j}$ is proportional to the rate at which such interaction accrues regardless of the state of individuals. That creates a total influx of $\sum _{j \in V} w_{ij} \pi _{j} (1-z_{i}(t))z_{j}.(t)$

The dynamics is given by the following system of ODEs:

1 $$\begin{aligned} \frac{\textrm{d}}{\textrm{d}t} z_{i}(t)=(1-z_{i}(t)) \sum _{j \in V} w_{ij} \pi _{j} z_{j}(t)-\gamma _{i} z_{i}(t) {\qquad (i\in V)}. \end{aligned}$$

The initial conditions are assumed to be $0 \le z_i(0) \le 1$ for all $i \in V$. Since the right hand side of (1) is locally Lipschitz-continuous, (1) has a unique solution. Furthermore, standard arguments show that the region $[0,1]^n$ is positive invariant (Delmas et al. 2020), that is,

$$\begin{aligned} 0 \le z_i(0) \le 1 \quad \forall i \in V \quad \Longrightarrow \quad 0 \le z_i(t) \le 1 \quad \forall t \ge 0 \,\forall i \in V, \end{aligned}$$

so $z_i(t)$ can indeed be interpreted as a ratio.

Using the notation $\mathcal {A}:=W \Pi = \left( a_{ij} \right) _{i,j \in V}$ and $z(t):= \left( z_{i}(t) \right) _{i \in V}$, we may rewrite (1) as

2 $$\begin{aligned} \frac{\textrm{d}}{\textrm{d}t} z_{i}(t)=(1-z_i(t)) \left( \mathcal {A} z(t) \right) _{i}-\gamma _i z_{i}(t)=:F_i^{\mathcal {A}}(z(t)) \qquad (i\in V). \end{aligned}$$

The *degree* of community $i \in V$ is defined as

3 $$\begin{aligned} d_i:= \sum _{j \in V} a_{ij} \end{aligned}$$

representing the maximal rate at which an individual at community $i \in V$ can get infected.

The system (1) is intimately related to the stochastic version of the SIS process on sufficiently dense simple graphs. Fix the parameters $n, W, \pi , \gamma ,$ and generate a random graph on *N* vertices where two vertices from communities $i,j \in V$ respectively are connected with probability (proportional to) $\kappa ^N w_{ij}$, where $\kappa ^N$ is a density parameter such that $\kappa ^N \gg \frac{ \log N}{ N}.$ Then, according to Keliger et al. (2022), the ratio of infected individuals in community $i \in V$ will stochastically converge to $z_{i}(t)$ uniformly on any compact time interval [0, *T*]. (Note that although the setting in Keliger et al. (2022) does not allow inhomogeneous $\gamma _i$ parameters directly, one can augment the state space to consist of pairs (*i*, *S*), (*i*, *I*) for different $i \in V$. Since *n* is fixed, the results in Keliger et al. (2022) remain valid for this state space as well.)

We point out that based on the Cauchy–Kovalevskaya theorem, $z_i(t)$ is also an analytic function of *t* as the right hand side of (1) is itself analytic. This property will be frequently exploited during the proofs, starting with the following lemma.

#### Lemma 2.1

Let $h: [0,\infty [ \rightarrow \mathbb {R}$ be an analytic function. Assume for some $T_0>0$ we have $\forall t \in [0,T_0] \ h(t)=0$. Then, $\forall t \ge 0 \ h(t)=0.$

The short proof can be found in Section 5.1.

We will make use of the Gram matrix

4 $$\begin{aligned} G_{ij}(T):= \int _{0}^{T}z_{i}(t) z_{j}(t) \textrm{d}t {\qquad (T>0).} \end{aligned}$$

It is symmetric, positive semi-definite, hence it induces a semi-scalar product

$$\begin{aligned} \Vert v \Vert _{G(T)}^2:= \langle v, G(T) v \rangle =\int _{0}^{T} \langle v, z(t) \rangle ^2 \textrm{d}t, \end{aligned}$$

where $\langle \cdot ,\cdot \rangle$ is the standard scalar product on $\mathbb {R}^n$.

$\Vert v \Vert _{G(T)}$ is zero on the nullspace of *G*(*T*), denoted by $\mathcal {N}.$ Lemma 2.1 applied for the analytic function $h_{{v}}(t):=\langle v, z(t) \rangle$ implies that for any $0<T_0 \le T$ the nullspaces are the same, hence, $\mathcal {N}$ does not depend on *T* as long as $T>0$.

We will also use the $L^r(V,\pi )$ norm:

$$\begin{aligned} \Vert v \Vert _{r,\pi }:= \left( \sum _{i \in V} \left| v_i \right| ^r \pi _i\right) ^{1/r}. \end{aligned}$$

Besides the induced matrix norm $\Vert \cdot \Vert _{p, \pi }$, we will also use the so-called cut norm defined as

5 $$\begin{aligned} \Vert W \Vert _{\square }:= \max _{S,S' \subseteq V} \left| \sum _{ \begin{array}{c} i \in S \\ j \in S' \end{array}}w_{ij }\pi _i \pi _j \right| . \end{aligned}$$

### The reconstruction problem

The setup for the reconstruction problem is the following. We have perfect knowledge of *z*(*t*) over a time interval $[0,T_0]$ ($T_0>0$), along with $\pi$ and $\gamma .$ The task is to find *W*. Reconstruction is impossible when $T_0=0$ as *W* can be chosen independently of *q*, hence, the reason for assumption $T_0>0$.

In practice, $\pi$ being available is a reasonable assumption since it only concerns the size of communities. Knowledge about $\gamma$ can be interpreted in several ways: it could be experimentally measured during clinical trials, or a different approach would be to assume that we do not only observe the total change of *z*(*t*) over time, but we also observe the gains $\left( \frac{\textrm{d}}{\textrm{d}t} z_{i}(t) \right) _{+}:= (1-z_{i}(t)) \left( \mathcal {A}z(t) \right) _{i}$ and losses $\left( \frac{\textrm{d}}{\textrm{d}t} z_{i}(t) \right) _{-}:=-\gamma _{i}z_{i}(t)$ separately, corresponding to new infections and recoveries respectively. From these, $\gamma _i$ can be expressed as

$$\begin{aligned} \gamma _{i}=- \frac{1}{z_{i}(t)} \left( \frac{\textrm{d}}{\textrm{d}t}z_{i}(t) \right) _{-}. \end{aligned}$$

We note that $\mathcal {A}z(t)$ is also an observable quantity since it has the form of

6 $$\begin{aligned} \left( \mathcal {A}z(t) \right) _{i}= \frac{\frac{\textrm{d}}{\textrm{d}t}z_{{i}}(t)+\gamma _iz_{i}(t)}{1-z_{i}(t)}. \end{aligned}$$

We set some technical restrictions on the parameters. In general, we want the weights $w_{ij}$ and the curing rates $\gamma _i$ to be bounded from above, and $z_i(t)$ to be bounded away from 1 (as $1-z_i(t)$ appears in the denominator of the right hand side of (6)). We also want $z_i(0), \pi _i$ and $w_{ij}$ to be bounded away from 0 to ensure a lower bound on the speed of the disease spreading (especially for the SI case). Accordingly, for bounds $0 <m \le 1/2$ and ${M>0}$, we define the parameter sets

7 $$\begin{aligned} \begin{aligned} \mathcal {Q}_{m,M}^{(n)}:=&\Bigg \{(z(0),\gamma ,\pi ) \in \mathbb {R}^n \times \mathbb {R}^n \times \mathbb {R}^n | \forall i \in V \ m \le z_{i}(0) \le 1-m, \\&0 \le \gamma _i \le M, 0\le \pi _i, \sum _{j\in V} \pi _j =1 \Bigg \}, \end{aligned}\end{aligned}$$

8 $$\begin{aligned} \mathcal {P}_{m,M}^{(n)}:=&\left\{ (W,q) \in \mathbb {R}^{n \times n} \times \mathcal {Q}_{m,M}^{(n)} \big | W^{\intercal }=W,\, \forall i,j \in V \, m \le w_{ij} \le M \right\} . \end{aligned}$$

#### Remark 2.2

For a fixed *n*, the assumption $m \le w_{ij} \le M$ is not too restrictive, but later on it will be used as $n \rightarrow \infty$ with $0<m \le M$ fixed, essentially making the graph sequence dense, enabling us to apply the theory of graphons in section 4. See also the proofs of Theorems 3.9 and 3.12.

For the reconstruction problem, we will always assume $\pi _i>0\, \forall i \in V$ as the dynamics is insensitive to vertices with $\pi _i=0$ (so reconstruction for those parts of the graph would be impossible anyway). Note that $\pi _i=0$ is not an issue for the prediction problem (see Section 2.3), where it is convenient to allow $\pi _i=0$ as *n* is allowed to increase.

Since (1) is well-posed, the parameters $p \in \mathcal {P}_{m,M}^{(n)}$ uniquely determine the trajectories $\left( z(t) \right) _{0 \le t \le T_0}$, hence we may define the mapping

$$\begin{aligned} \phi _{T_0}(p): \ p \mapsto \left( z(t) \right) _{0 \le t \le T_0}, \end{aligned}$$

that maps from $\mathcal {P}_{m,M}^{(n)}$ (for some fixed $0<m \le \frac{1}{2}, \ M>0$) to the space of analytical functions $([0,T_0] \rightarrow [0,1]^n).$

#### Definition 2.3

For $p=(W, q) \in \mathcal {P}_{m,M}^{(n)}$, we say *W* is *q-reconstructible* if for all $T_0>0$, the mapping $\phi _{T_0} \left( \cdot , q \right)$ is injective at *W*.

Not all *W* are reconstructible. For example, assume that the parameter *p* is regular in the sense that there exist $\bar{\gamma }, \bar{d}, \bar{z}(0)$ such that for all $i \in V$ we have $d_i=\bar{d}, \ \gamma _i=\bar{\gamma }$ and $z_{i}(0)=\bar{z}(0).$ It is easy to check that for all $i \in V$ the solution has the form of $z_i(t)=\bar{z}(t)$ where $\bar{z}(t)$ solves

$$\begin{aligned} \frac{\textrm{d}}{\textrm{d}t}\bar{z}(t)=\bar{d} (1-\bar{z}(t)) \bar{z}(t)-\bar{\gamma }\bar{z}(t). \end{aligned}$$

However, many other set of parameters $p \in \mathcal {P}_{m,M}^{(n)}$ satisfy the regularity assumption with the same triple ${( \bar{\gamma }, \bar{d}, \bar{z}(0))}$.

### The prediction problem

The prediction problem is the following: given *q* and the curve $\left( z(t) \right) _{0 \le t \le T_0}$, we aim to predict *z*(*t*) for $t>T_0$ assuming that $0<T_0 < T.$

One approach to the prediction problem is to find an approximate weighted graph $\hat{W}$ such that the corresponding solution $\hat{z}(t)$ is close to the original solution *z*(*t*) on $[0,T_0]$ according to some error measure, and use $\hat{z}(t)$ as a prediction for *z*(*t*) for $t>T_0$. In Prasse and Mieghem (2022) the authors constructed a $\hat{W}$ that is consistent with *z*(*t*) on $[0,T_0]$ by minimizing the error between $F^{\mathcal {A}}(z(t))$ and $F^{\hat{\mathcal {A}}}(z(t)).$ More precisely, they compute

$$\begin{aligned}&\operatorname {argmin}_{\hat{a}_{i,1}, \dots \hat{a}_{i,n}} \sum _{j=1}^{J} \left( \frac{\Delta z_i(t_j)}{\Delta t_j}-F_i^{\hat{\mathcal {A}}}(z(t_j)) \right) ^2+\rho _i \sum _{{k}=1}^{{n}} \hat{a}_{i{k}} \\&s.t. \ \hat{a}_{ij} \ge 0 \end{aligned}$$

for all $i \in V,$ where time is discretized into $0=t_0<t_1<\dots , <t_J=T_0$ and $\rho _i \ge 0$ serve as parameters to tune the relaxation terms.

We take a similar approach, but use a different error measure; instead of using the derivatives $F^{\mathcal {A}}(z(t))$ we make use of the neighborhood statistics $\mathcal {A}z(t)$ and $\hat{\mathcal {A}}z(t)$ ($\hat{\mathcal {A}}:=\hat{W} \Pi$) as these are also observable and have nicer analytical properties.

We introduce the $L^r\left( V, \pi \right)$ type error terms:

9 $$\begin{aligned} H_{r,T}(W,W'):= \left( \int _{0}^{T} \Vert \mathcal {A}z(t)-\hat{\mathcal {A}} z(t) \Vert _{r,\pi }^r \textrm{d}t \right) ^{1/r}. \end{aligned}$$

$H_{r,T_0}$ is an observable quantity but $H_{r,T}$ is not when $T>T_0$, as it depends on the future values of *z*(*t*). The following lemma states that if the error $H_{2,T}$ is small, then the trajectories generated by *W* and $\hat{W}$ stay close together on [0, *T*].

#### Lemma 2.4

Let $p, \hat{p} \in \mathcal {P}_{0,M}^{(n)}$ with the corresponding solutions of (1) being *z*(*t*) and $\hat{z}(t).$ We have

$$\begin{aligned} \sup _{0 \le t \le T} \Vert z(t)-\hat{z}(t) \Vert _{1,\pi } \le&\left( \Vert z(0)-\hat{z}(0) \Vert _{1,\pi }+T \Vert \gamma -\hat{\gamma } \Vert +H_{1,T} \right) e^{3MT}. \end{aligned}$$

Furthermore, assuming $\hat{z}(0)=z(0)$ and $\hat{\gamma }=\gamma$, we also have

$$\begin{aligned} \sup _{0 \le t \le T} \Vert z(t)-\hat{z}(t) \Vert _{1,\pi } \le e^{3MT}H_{1,T} \le \sqrt{T}e^{3MT} H_{2,T}. \end{aligned}$$

The proof can be found in Section 5.1.

Let $\eta ^{(i)}$ denote the *i*th row vector of $\mathcal {A}-\hat{\mathcal {A}}$ (that is, $\eta ^{(i)}_j:= a_{ij}-\hat{a}_{ij}).$ Then

$$\begin{aligned} \Vert \left( \mathcal {A}-\hat{\mathcal {A}} \right) z(t) \Vert _{2,\pi }^2=\sum _{i \in V} \langle \eta ^{(i)}, z(t) \rangle ^2 \pi _i, \end{aligned}$$

implying

10 $$\begin{aligned} H_{2,T}^2=\sum _{i \in V} \left\| \eta ^{(i)} \right\| _{G(T)}^2 \pi _i. \end{aligned}$$

Keeping all the parameters besides $\hat{W}$ fixed, the minimum of $H_{2,T_0}=H_{2,T_0}(\hat{W})$ as a function of $\hat{W}$ is 0, obtained at $\hat{W}=W.$ Using Lemma 2.1 for the function $h(t)=H_{2,t},$ we obtain $H_{2,T_0}=0 \ \Rightarrow H_{2,T}=0.$ This means that if we manage to find the minimum of $H_{2,T_0}$ without error, then perfect prediction is possible.

However, this is rarely possible; typically, we target $H_{2,T_0}$ to be smaller than some given threshold, and the task is then to show continuous dependence of the future error $H_{2,T}$ on the past error $H_{2,T_0}$, that is, we want to show that for all $\varepsilon >0$ there is a $\delta >0$ such that $H_{2,T_0}<\delta \Rightarrow H_{2,T}<\varepsilon .$

## Results

### Results for the reconstruction problem

We start by giving a characterization of *q*-reconstructability.

#### Theorem 3.1

Let $p=(W,q) \in \mathcal {P}_{m,M}^{(n)}$ and assume $\pi _i>0\, \forall i \in V.$

The following properties are equivalent:

1. *W* is *q*-reconstructible.
2. For any $$T_0>0$$, $$\forall t \in [0,T_0] \ \langle v, z(t) \rangle {=0} \Rightarrow v=0. $$
3. For any $T_0>0$, $G(T_0)$ is invertible.

*W* not being *q*-reconstructible does not necessarily mean that the counterexample $\hat{W}$ which generates the same trajectory also satisfies $(\hat{W},q)\in \mathcal {P}_{m,M}^{(n)}.$ That said, in the proof of Theorem 3.1, the $\hat{W}$ constructed satisfies $\hat{W} \sim \mathcal {P}_{m',M'}$ for arbitrary $0<m'<m \le M <M'$.

Reconstructability can also be understood in the literal sense, that is, there is an explicit formula for *W*. First define

$$\begin{aligned} R(T_0):= \int _{0}^{T_0} \mathcal {A} z(t) z^{\intercal }(t) \textrm{d}t. \end{aligned}$$

Both $G(T_0)$ (defined in (4)) and $R(T_0)$ are observable matrices which satisfy the identity

11 $$\begin{aligned} \mathcal {A}G(T_0)=R(T_0). \end{aligned}$$

According to Theorem 3.1, if *W* is *q*-reconstructible, then $G(T_0)$ is invertible, and

12 $$\begin{aligned} W=R(T_0)G^{-1}(T_0) \Pi ^{-1}. \end{aligned}$$

For any $\hat{\mathcal {A}}=\hat{W} \Pi$ satisfying $\hat{\mathcal {A}}G(T_0)=R(T_0)$, we also have

$$\begin{aligned} H_{2,T_0}^2(\hat{W})=&\int _{0}^{T_0} \sum _{i \in V} \left( \left( \mathcal {A}-\hat{\mathcal {A}} \right) z(t) \right) _i^2 \pi _i \textrm{d}t\\ =&\sum _{i \in V} \pi _i \Bigg (\underbrace{\int _{0}^{T_0} \left( \mathcal {A}-\hat{\mathcal {A}} \right) z(t) z^{\intercal }(t) \textrm{d}t }_{=R(T_0)-\hat{\mathcal {A}}G(T_0)=0}\left( \mathcal {A}-\hat{\mathcal {A}} \right) ^{\intercal } \Bigg )_{ii}=0, \end{aligned}$$

therefore, even if the solution to (11) is not unique (in the case when *W* is not *q*-reconstructible), any other solution gives the same trajectory as *W*.

None of the conditions of Theorem 3.1 are easy to check directly for the parameter *p*. We conjecture that there exists an equivalent condition that can be checked directly. In order to present it, we define block regularity. In plain words, block regularity means that it is possible to partition the blocks into communities, such that for any two communities from the same group have the same initial conditions, cure rates and degrees to all other partitions.

#### Definition 3.2

We call $p \in \mathcal {P}_{m,M}^{(n)}$ *block regular* if there is a partition $V_1,\dots , V_K$ of *V* for some $K<n$ such that

$$\begin{aligned}&\forall 1 \le k \le K{,} \ i_1,i_2 \in V_k \ z_{i_1}(0)=z_{i_2}(0), \ \gamma _{i_1}=\gamma _{i_2 }, \\&\forall 1 \le k,l \le K{,} \ i_1, i_2 \in V_k, \ \sum _{j \in V_l}a_{i_1j}=\sum _{j \in V_l} a_{i_2j}. \end{aligned}$$

#### Conjecture 3.3

Assuming $\forall i \in V \ \pi _i>0$ and $p=(W, q) \in \mathcal {P}_{m,M}^{(n)}$, *W* is *q*-reconstructible if and only if *p* is not block regular.

The only if part of Conjecture 3.3 is relatively straightforward. Let *p* be block regular and assume indirectly that it is reconstructible. The key insight is that block regular graphs can be reduced to a graph on *K* vertices. Fix $i \in V_k$. Define $\bar{z}_{k}(0):=z_{i}(0), \ \bar{\gamma }_k:=\gamma _i, \ \bar{\pi }_{k}:=\sum _{j \in V_k} \pi _j$ and

$$\begin{aligned} \bar{w}_{kl}:= \frac{1}{\bar{\pi }_k \bar{\pi }_l}\sum _{i \in V_k}\sum _{j \in V_l} w_{ij}\pi _i \pi _j . \end{aligned}$$

Clearly $\bar{p}:=\left( \bar{W}, \bar{z}(0), \bar{\gamma }, \bar{\pi } \right) \in \mathcal {P}_{m,M}^{(K)}.$ Furthermore, the solution *z*(*t*) has the following form:

$$\begin{aligned} \forall 1 \le k \le K, \ i \in V_{{k}}, t \ge 0 \ z_{i}(t)=\bar{z}_k(t), \end{aligned}$$

where $\bar{z}(t)$ solves (1) with parameter $\bar{p}.$ Choose $i_1,i_2\in V_k$, $i_1\ne i_2$, and let

$$\begin{aligned} v_j= { {1\!\!1}_{\left\{ j=i_1\right\} }-{1\!\!1}_{\left\{ j=i_2\right\} }.} \end{aligned}$$

Then clearly $\langle v, z(t)\rangle =z_{i_1}(t)-z_{i_2}(t)=0,$ so according to Condition 2 of Theorem 3.1, *W* is non-*q*-reconstructible.

We were not able to prove that non block regularity is a sufficient condition for reconstructability, except for populations with $n=2$ communities (The proof proof is left out of the article.) We did not manage to find any non reconstructible *p* which is not block regular. That being said, in the special case of the SI processes, there is a neat sufficient condition for reconstructability.

#### Theorem 3.4

For $q \in \mathcal {Q}_{m,M}^{(n)}$, assume the following properties:

- $\gamma =0$ (SI model), and
- $$\forall i \in V \ \pi _i>0$$, and
- the degrees $d_1, \dots , d_n$ are all different.

Then *W* is *q*-reconstructible.

#### Remark 3.5

If $\pi$ is uniform ($\pi _i= \frac{1}{n}$ for all $i \in V$) and *W* represents a simple unweighted graph, then there exist two equal degrees as the possible degrees are $0,1,\dots ,n-1$, but 0 and $n-1$ cannot be present simultaneously. This makes the last condition of Theorem 3.4 violated.

#### Remark 3.6

For any fixed $q \in \mathcal {Q}_{m,M}^{(n)}$ with $\gamma =0$ and $\forall i \in V, \pi _i>0$, the degrees are all different for almost every *W*, because $d_i=d_j$ ($i \ne j$) gives a lower dimensional subspace for *W*.

Lemma 6 in Beaufort et al. (2022) presents a result similar to Theorem 3.4, with some differences. Beaufort et al. (2022) studies the SIR process with diffusion and takes finitely many observations as opposed to full knowledge of the process over $[0,T_0].$ Most importantly though, the proof in Beaufort et al. (2022) relies on looking only at the total number of individuals at a given location, which only depends on the diffusion process but not on the epidemic dynamics, so the result is more about how to reconstruct the diffusion coefficients from the trajectories of the diffusion process rather than from the epidemic. The proof is also not applicable to the case when the diffusion is in equilibrium (even though that would be a natural assumption), while the result in the present paper is applicable for arbitrary initial conditions within $Q^{(n)}_{m,M}$.

#### Remark 3.7

In the SI model, the degrees are observable in the $t\rightarrow \infty$ limit due to

$$\begin{aligned} \lim _{t \rightarrow \infty } -\frac{1}{t} \log \left( 1- z_i(t) \right) =\lim _{t \rightarrow \infty } \frac{1}{t}\int _{0}^{t} \left( \mathcal {A}z(\tau )\right) _i \textrm{d}\tau -\frac{1}{t} \log \left( 1- z_{i}(0) \right) = \left( \mathcal {A} 1\!\!1 \right) _i=d_i. \end{aligned}$$

The relevance of this identity is that in Prasse and Mieghem (2022), one way to show that $\hat{W}$ significantly differs from *W* was to compare their degree distribution. However – at least for the SI case – if the error between *z*(*t*) and $\hat{z}(t)$ converges to 0, then the degree distribution of $\hat{W}$ also converges to the degree distribution of *W*.

While Theorem 3.4 states that for any given *q*, almost every *W* is *q*-reconstructible (at least in the SI case), it does not imply that the reconstruction problem is robust. Indeed, if we take a sequence $p_N \rightarrow p$ where $p=(W,q)$ is such that *W* is non-*q*-reconstructible, then (11) becomes more and more ill-conditioned as $N \rightarrow \infty$.

The reconstruction problem becomes harder as we let $n \rightarrow \infty$. We introduce a further definition: a special case of block regular parameters are block homogeneous ones. The main difference between the two concepts is that block homogeneous networks required that the edge weights should be the same between the same type of blocks, while in the case of block regularity, it is enough that only the incoming block degrees are identical.

#### Definition 3.8

$p \in \mathcal {P}_{m,M}^{(n)}$ is *block homogeneous* if it is block regular with $K<n$ blocks and there exist $m \le \bar{w}_{kl} \le M$ ($1 \le k, l \le K$) such that it satisfies

$$\begin{aligned} \forall 1 \le k, l \le K, i \in V_k, j \in V_l{,} \ w_{ij}=\bar{w}_{kl}. \end{aligned}$$

Szemerédi’s regularity lemma essentially states that any $p \in \mathcal {P}^{(n)}_{m,M}$ can be approximated by some block homogeneous $\hat{p} \in \mathcal {P}_{m,M}^{(n)}$ corresponding to some lower-dimensional parameter $\bar{p} \in \mathcal {P}_{m,M}^{(K)}$ where $K \ll n.$

#### Theorem 3.9

For every $\varepsilon >0$ there is an $n_0$ such that for all $n \ge n_0$ and $p=(W,q) \in \mathcal {P}_{m,M}^{(n)}$ with $\pi _i=\frac{1}{n}$ there is a $\hat{W}$ such that

$$\begin{aligned}&\Vert W-\hat{W} \Vert _{\square } \ge \frac{1}{16}m>0 \\&\sup _{0 \le t \le T} \Vert z(t)-\hat{z}(t)\Vert _{1, \pi }<\varepsilon . \end{aligned}$$

#### Remark 3.10

The condition $\pi _i= \frac{1}{n}$ is mainly for convenience. However, some regularity about the population size must be assumed. As a counterexample, consider $\pi _{1}=1-\frac{1}{n-1}$ and $\pi _{i}=\frac{1}{(n-1)^2}$ for $i>1$. In this setting, the problem is basically reduced to a weighted graph with one vertex where the only relevant parameter from *W* is $w_{11}$, corresponding to the infection rate of the main block.

### Results for the prediction problem

Let $v \in \mathbb {R}^n$ be arbitrary and $u \in \mathcal {N}^{\intercal }$ be its orthogonal projection that is orthogonal to the nullspace $\mathcal {N}$. Clearly, $\Vert v\Vert _{G(T)}=\Vert u \Vert _{G(T)}$ for all $T>0.$ Notice that $\mathcal {N}^{\intercal }$ is a finite dimensional subspace on which both $\Vert \cdot \Vert _{G(T)}$ and $\Vert \cdot \Vert _{G(T_0)}$ are norms. Since on finite dimensional spaces all norms are equivalent, there is a constant $\Lambda ^{(n)}(p)$ such that

$$\begin{aligned} \Vert u \Vert _{G(T)} \le \Lambda ^{(n)}(p) \Vert u\Vert _{G(T_0)}\quad \forall u \in \mathcal {N}^{\intercal }, \end{aligned}$$

implying

13 $$\begin{aligned} H_{2,T} \le \Lambda ^{(n)}(p) H_{2,T_0} \end{aligned}$$

based on (10). (Note that $\Lambda ^{(n)}(p)$ also depends on $T_0,T,m,M$ and $\pi$.) This means that for any *fixed* $p \in \mathcal {P}_{m,M}^{(n)}$ prediction is possible, and we also have $H_{2,T}=O \left( H_{2,T_0} \right) .$

However, (13) is weak in the sense that the dependence on *p* is not specified. For example, the previous argument does not exclude the possibility that for some sequence $p_N \rightarrow p^*$ ($N \rightarrow \infty$) we have $\Lambda ^{(n)}\left( p_N\right) \rightarrow \infty$ even though $\Lambda ^{(n)}(p^*)<\infty$. ($\Lambda ^{(n)}(p)$ is not continuous in *p*.) We conjecture this not to be the case for the prediction problem.

#### Conjecture 3.11

14 $$\begin{aligned} \Lambda ^{(n)}:=&\sup _{p \in \mathcal {P}^{(n)}_{m,M}} \Lambda ^{(n)}(p)<\infty \end{aligned}$$

15 $$\begin{aligned} \Lambda :=&\sup _{n { \in \mathbb {N}^+}} \Lambda ^{(n)}<\infty \end{aligned}$$

A slightly weaker yet still surprisingly strong statement shows, however, that for $H_{2,T_0}<\delta \Rightarrow H_{2,T}<\varepsilon$ we can choose $\delta$ independently of both $\mathcal {P}_{0,M}^{(n)}$ and *n*. This means the prediction problem can be solved very robustly even for large dimension.

#### Theorem 3.12

Assuming $0 \le \hat{w}_{ij} \le M$ ($i,j \in V$) For all $\varepsilon >0$ there is a $\delta >0$ such that $H_{2,T_0}<\delta \Rightarrow H_{2,T}<\varepsilon$, where the choice of $\delta >0$ is uniform in $p \in \bigcup _{n \in \mathbb {N^{+}}}\mathcal {P}_{0,M}^{(n)}$.

### Discrete time and measurement errors

So far, we assumed that *z*(*t*) is available for all $0\le t\le T_0$ with arbitrary precision. In real-life scenarios, this is typically not the case; the values of *z*(*t*) are only available at certain measurement points, and the $z(t_j)$ values may also have measurement errors. This section addresses the prediction error resulting from only knowing *z*(*t*) at the discrete measurement points, and also the error resulting from measurement error.

The measurement points are denoted by $0=t_0< t_1< \dots < t_J = T_0$, and the measurement values are $z^*(t_i), i=0,\dots , J.$ Each $z^*(t_i)$ is a vector of length *n*. Technically, $z^*(\cdot )$ is only defined at the $t_0,t_1,\dots ,t_J$ points, but we will consider it extended to $[0,T_0]$ as a piecewise constant function between the measurement points. $\gamma$ is also measured with error; the corresponding measurement is denoted by $\gamma ^*\in \mathbb {R}^n$.

Since it is convenient to use the $L^2(V,\pi )$ norm, for the sake of simplicity, we do not account for the the measurement error of $\pi$. Note that, in practice, the distribution of the population is easy to estimate compared to all other parameters. It is possible to prove continuous dependence on $\pi$, yet it would yield scant insight.

#### Remark 3.13

For $p \in \mathcal {P}_{m,M}^{(n)}$ the largest infection rate a population can receive is *M*. Combining with the condition $1-z_i(0) \ge m,$ this implies that the ratio of *susceptible* individuals is bounded bellow by

$$\begin{aligned} 1-z_i(t) \ge m e^{-MT_0} \ \ \left( \forall i \in V, t \in [0,T_0] \right) . \end{aligned}$$

Using Remark 3.13, without the loss of generality, we may assume the same for the estimated values $z^*(t)$, that is

16 $$\begin{aligned} 1-z_i^*(t) \ge m e^{-MT_0} \ \ \left( \forall i \in V, t \in [0,T_0] \right) . \end{aligned}$$

Even if we measure a value bellow $m e^{-MT_0}$ in practice, we could replace our estimate $1-z_i^*(t)$ with $m e^{-MT_0}$ (or even $me^{-Mt}$) as it would result in a lower error due to our a prior knowledge. We will make this assumption throughout the rest of the paper.

We introduce the error terms

$$\begin{aligned} \Delta _1&:= \Vert z(0)-z^*(0)\Vert _{\pi ,2},\\ \Delta _2&:= \Vert \gamma -\gamma ^*\Vert _{\pi ,2},\\ \Delta _3&:= \max _{1\le i\le J}\frac{1}{t_i-t_{i-1}} { \left\| \left[ z(t_i)-z(t_{i-1})\right] -\left[ z^*(t_i)-z^*(t_{i-1}) \right] \right\| _{\pi ,2}},\\ \Delta _4&:= \max _{1\le i\le J} (t_i-t_{i-1}). \end{aligned}$$

Condition (16) is motivated by the term $1-z_j^*(t_i)$ in the denominator of (17), which is now guaranteed to be separated from 0. Note that $\Delta _3$ essentially corresponds to the measurement error of $\frac{\textrm{d}z(t)}{\textrm{d}t}$; it does not necessarily decrease if we increase the number of measurements, creating an interesting tension with $\Delta _4$.

To keep the derivations shorter, discrete measurement points and measurement error will be handled simultaneously:

$$\begin{aligned} \Delta :=\Delta _1+\Delta _2+\Delta _3+\Delta _4, \end{aligned}$$

and we assume that we can make measurements frequently and accurately enough to make $\Delta$ small. Motivated by (6), we define

17 $$\begin{aligned} \alpha _j(t_i):=\frac{\frac{z_j^*(t_i)-z_j^*(t_{i-1})}{t_i-t_{i-1}}+\gamma _j^*z_j^*(t_i)}{1-z_j^*(t_i)} \end{aligned}$$

and

18 $$\begin{aligned} (H_{2,T_0}^*(\hat{W}))^2:= \sum _{i=1}^J\sum _{j=1}^n |\alpha _j(t_i)-(\hat{\mathcal {A}}z^*(t_i))_j|^2\pi _j (t_i-t_{i-1}). \end{aligned}$$

The main result of this section is the following:

#### Theorem 3.14

Assume $p \in \mathcal {P}_{m,M}^{(n)}$, $m \le \hat{w}_{ij} \le M$ and (16). We have that

19 $$\begin{aligned} (H_{2,T_0}^*(\hat{W}))^2=H_{2,T_0}^2(\hat{W})+O(\Delta ). \end{aligned}$$

(The $O(\cdot )$ depends on $m,M,T_0$, but not on *n*.)

## Setup for graphons

In scenarios where *n* is allowed to vary, it is convenient to work in a state space that can incorporate weighted graphs of different sizes. Let each vertex *i* be represented by a subset $I_i \subseteq [0,1]$ with $\left| I_i \right| =\pi _i.$ such that $I_1, \dots I_n$ forms a partition of [0, 1]. This way, we may lift the weighted graph $W= \left( w_{ij} \right) _{ij \in V}$ up to the space of symmetric (measurable) kernel functions $m \le W(x,y)=W(y,x) \le M$ via

$$\begin{aligned} W(x,y):=\sum _{i, j \in V}w_{ij} {1\!\!1}_{\left\{ x \in I_i\right\} } {1\!\!1}_{\left\{ y \in I_j \right\} }, \end{aligned}$$

or in other words, we set $W(x,y)=w_{ij}$ when $x \in I_i$ and $y\in I_j$

Note that originally the notation *W* referred to the matrix $\left( w_{ij} \right) _{i,j \in V}$. With some light abuse of notation, we will also write *W* to denote the kernel function $W(\cdot , \cdot )$. $\hat{W}(x,y)$ is an analogous extension of the matrix $\hat{W}$.

Measurable kernels satisfying $0 \le W(x,y) =W(y,x) \le 1$ (a.e. $x,y \in [0,1]$) are called graphons in the literature and they arise as the natural limit objects of dense graph sequences (Lovász 2012). With a slight abuse of terminology, we will call any bounded, non-negative symmetric kernel graphons as well as they can always be rescaled. The corresponding integral operator is defined as

$$\begin{aligned} \mathbb {W}f(x):=\int _{0}^{1} W(x,y) f(y) \textrm{d}y. \end{aligned}$$

The definition of $\hat{\mathbb {W}}$ is analogous.

Similarly, the solution $(z_i(t))_{i \in V}$ is also lifted up to the space of (measurable) $\mathbb {R}_0^+ \times [0,1] \rightarrow [0,1]$ functions via

$$\begin{aligned} u(t,x):=\sum _{i \in V}z_{i}(t) {1\!\!1}_{\left\{ x \in I_i\right\} }, \end{aligned}$$

while the recovery rates $(\gamma _i)_{i \in V}$ become

$$\begin{aligned} \gamma (x):=\sum _{i \in V} \gamma _i {1\!\!1}_{\left\{ x \in I_i\right\} }. \end{aligned}$$

If $\left( z_i(t) \right) _{i \in V}$ is a solution to (1), then *u*(*t*, *x*) is a solution to

20 $$\begin{aligned} \partial _tu(t,x)=(1-u(t,x)) \mathbb {W}u(t,x)-\gamma (x) u(t,x). \end{aligned}$$

Define the extended parameter set to a set of measurable functions satisfying

21 $$\begin{aligned} \begin{aligned} \mathcal {P}_{m,M}:=&\big \{(W,u(0),\gamma ) | m \le W(x,y) =W(y,x) \le M,\\ &m \le u(0,x) \le 1-m, 0 \le \gamma (x) \le M, \textit{a.e } x,y \in [0,1] \big \}. \end{aligned} \end{aligned}$$

For any parameter $p \in \mathcal {P}_{m,M}$, there is a solution *u*(*t*) to (20) defined for all $t \ge 0$ such that $0 \le u(t,x) \le 1$ (see Delmas et al. (2020)).

We will call a parameter $p \in \mathcal {P}_{m,M}$ *discrete* if it corresponds to some finite interval partition $I_1, \dots , I_n$, in which case *p* can be identified as an element of $\mathcal {P}_{m,M}^{(n)}.$ The extension of (9) for graphons is

22 $$\begin{aligned} H_{r,T}:= \left( \int _{0}^{T} \left\| Wu(t)-\hat{W}u(t) \right\| _r^r \textrm{d}t \right) ^{1/r}, \end{aligned}$$

which coincides with (9) for the discrete case. The cut norm of a graphon *W* is defined as

23 $$\begin{aligned} \Vert W \Vert _{\square }:= \sup _{S,S' \subseteq [0,1]} \left| \int _{S} \int _{S'} W(x,y) \textrm{d}x \textrm{d}y \right| \end{aligned}$$

where the supremum is taken over measurable sets.

### Remark 4.1

Assume *W* is discrete. Let $S_i:=S \cap I_i, \ S_i':=S' \cap I_i$ and $x_i:=|S_i|, y_i:=|S_i'|$. Clearly $0 \le x_i,y_i \le \pi _i.$

Define

$$\begin{aligned} f(x,y):=x^{\intercal } Wy=\sum _{i,j} w_{ij}|S_i| |S'_j|=\int _{S} \int _{S'} {W(x',y') \textrm{d}x' \textrm{d}y'}. \end{aligned}$$

Assume that (*x*, *y*) either maximizes (or minimizes) *f*. Then $\frac{\partial f}{\partial x_i}= (Wy)_i=0$ for any $0<x_i<\pi _i$, implying

$$\begin{aligned} f(x,y)=x^{\intercal }Wy=\sum _{0<x_i<\pi _i} x_i (Wy)_i+\sum _{x_i=\pi _i}x_i (Wy)_i=\sum _{x_i=\pi _i}x_i (Wy)_i. \end{aligned}$$

This means that for any *i* for which $0<x_i<\pi _i$, the same maximum (or minimum) value of *f* can be obtained by setting $x_i=0$. Thus, without loss of generality, we may assume that for all *i* either $S_i=I_i$ or $S_i= \emptyset .$

The same argument applies for $S'$ as well, hence, in the discrete case, (23) and (5) are consistent. Actually, in this context, assuming *W* nonnegative is not necessary, which is useful when *W* is chosen as $W=W_1-W_2$, where $W_1,W_2$ are nonnegative.

We extend the notion of the cut norm to the parameter set $\mathcal {P}_{m,M}$ as

24 $$\begin{aligned} \Vert p \Vert _{\square }:= \Vert W \Vert _{\square }+ \Vert u(0) \Vert _1+ \Vert \gamma \Vert _1. \end{aligned}$$

Note that for *u*(0) and $\gamma$, the $L^1([0,1])$ norm is used; in 1-dimension, it is equivalent to the cut norm.

A slight generalization of Lemma 2.4 yields

### Lemma 4.2

$$\begin{aligned} \sup _{0 \le t \le T} \Vert u(t)-\hat{u}(t) \Vert _1 \le \left( \Vert u(0)-\hat{u}(0) \Vert _1+T\Vert \gamma -\hat{\gamma } \Vert _1+H_{1,T} \right) e^{3MT}. \end{aligned}$$

### Remark 4.3

Take a sequence $(p_N) \subset \mathcal {P}_{m,M}$ such that $p_N \overset{\Vert \cdot \Vert _{\square }}{\rightarrow }p.$ This clearly implies $u_N(0) \overset{L^1([0,1])}{\rightarrow }\ u(0)$ and $\gamma _N \overset{L^1([0,1])}{\rightarrow }\ \gamma$ (using obvious notation for the sequences $u_N(0)$ and $\gamma _N$).

Define $V_N:=\frac{1}{M}(W_N-W)$ and the corresponding integral operator $\mathbb {V}_N:= \frac{1}{M}(\mathbb {W}_N-\mathbb {W}).$ Since $|V_n| \le 1$, we may use the inequality

$$\begin{aligned} \Vert \mathbb {V}_N \Vert _{2} \le \sqrt{8 \Vert V_N \Vert _{\square } } \rightarrow 0 \end{aligned}$$

from Ruiz et al. (2024), implying

$$\begin{aligned} H_{1,T}^2(W_N) \le T H_{2,T}^2(W_N)=T M^2 \int _{0}^{T} \Vert \mathbb {V}_N u(t) \Vert _2^2 \textrm{d}t \le T^2 M^2 \Vert \mathbb {V}_N \Vert _2^2 \rightarrow 0, \end{aligned}$$

resulting in $u_N(t) \overset{L^1([0,1)]}{\rightarrow }u(t)$ uniformly in $t \in [0,T].$

The graphon extension of Szemerédi’s regularity lemma (László and Szegedy 2007) shows that any graphon can be approximated up to some error $\varepsilon >0$ with a discrete graphon with at most $K_{\max }(\varepsilon )$ blocks. The proof can be extended to the parameter set $\mathcal {P}_{m,M}$ by minor changes.

### Lemma 4.4

(Szemerédi’s lemma for $\mathcal {P}_{m,M}$)

$\forall \varepsilon >0 \ \exists K_{\max }(\varepsilon )\ \forall p \in \mathcal {P}_{m,M} \ \exists p' \in \mathcal {P}_{m,M}$ with the following properties:

1. $$\Vert p-p' \Vert _{\square }<\varepsilon $$,
2. $p'$ is discrete with partition $I_1', I_2', \dots , I_K'$ such that $1 \le K \le K_{\max }(\varepsilon )$ and
  $$\begin{aligned} w_{kl}':=&\frac{1}{\pi _k' \pi _l'} \int _{I_k} \int _{I_l}W(x,y) \textrm{d}x \textrm{d}y \\ z'_k(0):=&\frac{1}{\pi _k} \int _{I_k} u(0,x) \textrm{d}x \\ \gamma _k':=&\frac{1}{\pi _k} \int _{I_k} \gamma (x) \textrm{d}x, \end{aligned}$$
3. when *p* is discrete with partition $I_1, \dots , I_n$ such that $n \ge K_{\max }(\varepsilon )$ then the partition $(I_k)_{k=1}^{K}$ can be chosen in a way that $(I_i)_{i=1}^n$ is a refinement of $(I_k)_{k=1}^{K}.$

### Remark 4.5

From 3) it is easy to see that $p'$ is block homogeneous when *p* is discrete.

One issue with the cut norm is that it depends on the labeling of the vertices and it might distinguish between two isomorphic graphs. Instead, we might want to consider unlabeled graphs where the permutation of vertices are treated as equivalent. The graphon analogy of permutations are bijective measure preserving mappings $\phi : [0,1] \rightarrow [0,1]$ forming the set

$${ \Phi =\left\{ \phi \big |\phi : [0,1] \rightarrow [0,1], \phi \text { bijective, } \mathcal {L}(\phi ^{-1}(A))=\mathcal {L}(A) \text { for all }A \text { Borel sets} \right\} }.$$

The corresponding change of coordinates ("relabeling") of one and two variable functions *f*(*x*), *g*(*x*, *y*) are denoted by

$$\begin{aligned} f^{\phi }(x):=f(\phi (x)),\qquad g^{\phi }(x,y):=g(\phi (x),\phi (y)). \end{aligned}$$

Similarly, for $p \in \mathcal {P}_{m,M}$, we use the convention $p^{\phi }:=(W^{\phi },(u(0))^{\phi },\gamma ^{\phi })$. It is easy to check that $\Vert p^{\phi } \Vert _{\square }=\Vert p\Vert _{\square }.$

### Remark 4.6

Relabeling only effects the solution of (20) up to a change of coordinates:

$$\begin{aligned} \partial _t u^{\phi }(t,x)=&\partial _t u(t,\phi (x))=(1-u(t,\phi (x))\int _{0}^{1}W(\phi (x),y) u(t,y) \textrm{d}y \\&-\gamma (\phi (x))u(t,\phi (x)) \\ =&(1-u^{\phi }(t,x))\int _{0}^{1}W^{\phi }(x,y)u^{\phi }(t,y) \textrm{d}x-\gamma ^{\phi }(x)u^{\phi }(t,{y}) \end{aligned}$$

corresponding to the solution for the parameter $p^{\phi }.$

We define an equivalence relation between $p,p' \in \mathcal {P}_{m,M}$ where $p \sim p'$ if and only if there is a $\phi \in \Phi$ such that $p^{\phi }=p'$ a.e. The factorized space is denoted by $\tilde{\mathcal {P}}_{m,M}:= \mathcal {P}_{m,M} / \sim$ on which we define the (analogue of the) cut distance:

$$\begin{aligned} d_{\square }(p,p'):=\inf _{ \phi \in \Phi } \left\| p^{\phi }-p' \right\| _{\square }. \end{aligned}$$

### Remark 4.7

Clearly $d_{\square }(p,p') \ge 0$ with equality if and only if $p \sim p'.$ Symmetry also holds due to

$$\begin{aligned} \left\| p^{\phi }-p' \right\| _{\square }=\left\| p-\left( p'\right) ^{\phi ^{-1}} \right\| _{\square }. \end{aligned}$$

For the triangle inequality, we note that for any $\varepsilon >0$ we may choose $\phi _1,\phi _2 \in \Phi$ such that

$$\begin{aligned} \left\| p^{\phi _1}-p' \right\| _{\square } \le d_{\square }(p,p')+\varepsilon , \qquad \Vert p'-(p'')^{\phi _2} \Vert _{\square } \le d_{\square }(p',p'')+\varepsilon . \end{aligned}$$

Then

$$\begin{aligned} d_{\square }(p,p'')&\le \left\| p^{\phi _1 \circ \phi _2^{-1}} -p''\right\| _{\square }=\left\| p^{\phi _1 } -(p'')^{\phi _2}\right\| _{\square } \\&\le \left\| p^{\phi _1}-p' \right\| _{\square }+\Vert p'-(p'')^{\phi _2} \Vert _{\square } \le d_{\square }(p,p')+d_{\square }(p',p'')+2 \varepsilon . \end{aligned}$$

Letting $$\varepsilon \rightarrow 0$$ gives

$$\begin{aligned} d_{\square }(p,p'')&\le d_{\square }(p,p')+d_{\square }(p',p''). \end{aligned}$$

### Lemma 4.8

The metric space $\left( \tilde{\mathcal {P}}_{m,M}, d_{\square } \right)$ is compact.

The martingale argument in the proof of Theorem 5.1 in László and Szegedy (2007) applies for the proof of Lemma 4.8 with straightforward modifications.

## Proofs

### Proofs of general statements

#### Proof of Lemma 2.1

Define $T:= \sup \{t \ge 0 | h(t)=0 \}.$ Clearly, $0<T_0 \le T$. Indirectly assume $T<\infty .$ For all $0 \le t<T$ we have $h(t)=0$, which means all the left derivatives of *h* are 0 at *T*, hence, the Taylor series expansion is 0 around *T*. Then there exists an $\varepsilon >0$ such that $h(t)=0 \, \forall t \in [T,T+\varepsilon ].$ This would imply $T +\varepsilon \le T$, resulting in a contradiction. $\square$

Next, we prove Lemma 4.2 which directly implies Lemma 2.4.

#### Proof of Lemma 4.2

The proof follows simple Grönwall-type argument.

$$\begin{aligned} u(t,x)=&\, u(0,x)+\int _{0}^{t} \left( 1-u(\tau ,x) \right) \mathbb {W}u({\tau },x)-\gamma (x) u(\tau ,x) \textrm{d}\tau \end{aligned}$$

and similarly for $\hat{u}$; then

$$\begin{aligned} \left| u(t,x)-\hat{u}(t,x) \right| \le&\left| u(0,x)-\hat{u}(0,x) \right| +\int _{0}^{t} \underbrace{\left| \mathbb {W}u(\tau ,x) \right| }_{\le M} \cdot \left| u(\tau ,x)-\hat{u}(\tau ,x) \right| \textrm{d}\tau +\\&\int _{0}^{t} \underbrace{\left| 1-\hat{u}(\tau ,x) \right| }_{\le 1} \cdot \left| \mathbb {W}u(\tau ,x)- \hat{\mathbb {W}}\hat{u}(\tau ,x) \right| \textrm{d}\tau + \\&\int _{0}^{t} \underbrace{\gamma (x)}_{ \le M} \left| u(\tau ,x)-\hat{u}(\tau ,x) \right| \textrm{d}\tau + \int _{0}^{t} \underbrace{\hat{u}(\tau ,x)}_{ \le 1} \left| \gamma (x)-\hat{\gamma }(x)\right| \textrm{d}\tau . \end{aligned}$$

This gives

$$\begin{aligned} \left| u(t,x)-\hat{u}(t,x) \right| \le&\left| u(0,x)-\hat{u}(0,x) \right| +T \left| \gamma (x)-\hat{\gamma }(x) \right| \\&+\int _{0}^{t} \underbrace{\left| \hat{\mathbb {W}} \left[ u(\tau ,x)-\hat{u}(\tau ,x) \right] \right| }_{ \le M \left\| u(\tau )-\hat{u}(\tau ) \right\| _1} \textrm{d}\tau + \\&\int _{0}^{t} \left| \left[ \mathbb {W}-\hat{\mathbb {W}} \right] u(\tau ,x) \right| \textrm{d}\tau +2M \int _{0}^{t} \left| u(\tau ,x)-\hat{u}(\tau ,x)\right| \textrm{d}\tau \end{aligned}$$

which in turn gives

$$\begin{aligned} \Vert u(t)-\hat{u}(t) \Vert _{1} \le&\Vert u(0)-\hat{u}(0) \Vert _{1}+T \Vert \gamma -\hat{\gamma } \Vert _{1}+\underbrace{\int _{0}^{t} \left\| \left( \mathbb {W}-\hat{\mathbb {W}} \right) u({\tau })\right\| _{1}}_{=H_{1,t} \le H_{1,T} } + \\&3M \int _{0}^{t} \Vert u(\tau )-\hat{u}(\tau ) \Vert _{1 } \textrm{d}\tau . \end{aligned}$$

Finally, applying Grönwall’s Lemma gives

$$\begin{aligned} \sup _{0 \le t \le T}\Vert u(t)-\hat{u}(t) \Vert _{1} \le&\left( \Vert u(0)-\hat{u}(0) \Vert _{1}+T \Vert \gamma -\hat{\gamma }\Vert _{1}+H_{1,T} \right) e^{3MT}. \end{aligned}$$

(And the second statement of Lemma 2.4 also follows straightforward.) $\square$

#### Proof of Lemma 4.4

To extend Szemeredi’s lemma for the triple $(W, u(0),\gamma )$ we approximate *u*(0) and $\gamma$ by discretizing their range [0, 1] and [0, *M*].

From László and Szegedy (2007), for any *W* and $\varepsilon >0$ there exists a $W'$ such that $\Vert W-W' \Vert _{\square }<\varepsilon$, and $\tilde{W}$ is constant over some intervals $I_1', \dots , I_{K'}'$, with $K' \le 2^{ \lceil \frac{2}{\varepsilon ^2} \rceil }$. Furthermore, when *W* is discrete with partition $I_1,\dots , I_n$, we can choose $(I_k')_{k=1}^{K'}$ so that $(I_i)_{i=1}^{n}$ is a refinement of $(I_k')_{k=1}^{K'}$ if $n \ge 2^{ \lceil \frac{2}{\varepsilon ^2} \rceil }.$

In the next step, we construct $u'(0).$ Since $0 \le u(0,x) \le 1$, we may use

$$\begin{aligned} I_k'':= \left\{ x \in [0,1]\, | \, \frac{k-1}{K''} \le u(0,x)<\frac{k}{K''} \right\} \end{aligned}$$

where $K'':= \lceil \frac{1}{\varepsilon } \rceil$; apart from $\frac{k-1}{K''} \le u'(0,x) \le \frac{k}{K''}$, the value of $u'(0,x)$ is arbitrary. Clearly $\Vert u(0)-u'(0) \Vert _1 \le \varepsilon .$

Similarly, $0 \le \gamma (x) \le M$, hence, the same argument provides a $\gamma '$ with partition $I_1''', \dots , I_{K'''}'''$.

Take the smallest common refinement of $(I_k)_{k=1}^{K'}, (I_k)_{k=1}^{K''}, (I_k)_{k=1}^{K'''}$ and denote it by $(I_k)_{k=1}^{K}.$ We define $W'$ so that

$$\begin{aligned} w_{kl}':=\frac{1}{\pi _k' \pi _l'} \int _{I_k} \int _{I_l}W(x,y) \textrm{d}x \textrm{d}y; \end{aligned}$$

according to László and Szegedy (2007); Alon et al. (2002),

$$\begin{aligned} \Vert W-W' \Vert _{\square } \le 2 \varepsilon . \end{aligned}$$

We now specify the values of $u'(0)$ and $\gamma '$ as

$$\begin{aligned} u'_k(0):=&\frac{1}{\pi _k} \int _{I_k} u(0,x) \textrm{d}x, \\ \gamma _k':=&\frac{1}{\pi _k} \int _{I_k} \gamma (x) \textrm{d}x, \end{aligned}$$

resulting in

$$\begin{aligned} \Vert p-p' \Vert _{\square }=\Vert W-W' \Vert _{\square }+\Vert u(0)-u'(0) \Vert _{1}+ \Vert \gamma -\gamma ' \Vert _{1} \le 4 \varepsilon . \end{aligned}$$

(For simplicity, we settle for $4\epsilon$ as the final bound instead of $\epsilon$ as in the original statement of Lemma 4.4, but the lemma is obviously valid for either.) $\square$

### Proof of Section 3.1

#### Proof of Theorem 3.1

Since the nullspace of $G(T_0)$ is composed of vectors $v \in {\mathbb {R}^n}$ such that

$$\begin{aligned} \int _{0}^{T_0} \langle v, z(t) \rangle ^2 \textrm{d}t =0, \end{aligned}$$

2 $$\iff $$ 3 is trivial.

Now assume there exists a vector $0 \ne v \in \mathcal {N}.$ Set $u_i:= \frac{v_i}{\pi _i}$ and $\hat{w}_{ij}:=w_{ij}-\varepsilon u_iu_j.$ Clearly, $\hat{W}^{\intercal }=\hat{W}$ and we can choose $\varepsilon >0$ to be small enough such that $m' \le \hat{w}_{ij} \le M'$ for any $0<m'<m \le M <M'$ making $\hat{W} {\in } \mathcal {P}_{m',M'}^{(n)}.$

$$\begin{aligned} \eta ^{(i)}_j=&\, \varepsilon u_i u_j \pi _j=\varepsilon u_i v_j \Rightarrow \eta ^{(i)}=\varepsilon u_i v ,\\ \left\| \eta ^{(i)} \right\| _{G(T_0)}^2=&\, \varepsilon ^2 u_i^2 \underbrace{\Vert v \Vert _{G(T_0)}^2}_{=0}=0 \ \overset{(10)}{\Rightarrow }\ H_{2,T_0}=0. \end{aligned}$$

$H_{2,T_0}=0$ and Lemma 2.4 implies $z(t)=\hat{z}(t)$ for all $0 \le t \le T_0$ while $\hat{W} \ne W,$ which means *W* is not *q*-reconstructible.

Lastly, assume $\mathcal {N}= \{0 \}$ making $\Vert \cdot \Vert _{G(T_0)}^2$ a norm. Let $\hat{W}$ be a network such that $\hat{z}(t)=z(t)$ for $t \in [0,T_0].$ Due to (6) for $t \in [0,T_0]$ we also have $\mathcal {A}z(t)=\hat{\mathcal {A}}\hat{z}(t)= \hat{\mathcal {A}}z(t)$, implying $H_{2,T_0}=0.$ Consequently, (10) yields

$$\begin{aligned} \forall i \in V \ \Vert \eta ^{(i)} \Vert _{G(T_0)}^2=0 \quad \Rightarrow \quad \forall i \in V \ \eta ^{(i)}=0 \quad \Rightarrow \quad \hat{\mathcal {A}}=\mathcal {A} \quad \Rightarrow \quad \hat{W}=W, \end{aligned}$$

making *W* *q*-reconstructible. $$\square $$

#### Proof of Theorem 3.4

The main idea of the proof is the following: For the SI process the equilibrium state is when all regions are fully infected. Near said equilibrium, a susceptible individual at community *i* would perceive all of its neighbors to be infected, thus it gets infected at rate $d_i$.

The decay of the remaining susceptible vertices is the slowest in the community with the smallest degree, say $d_1$. Therefore, the leading term in $\langle v, 1-z(t) \rangle$ is proportional to $v_1$. This shows us that $v_1$ must be 0. By eliminating the community with the smallest degree, we can apply the same logic for the rest of the population, showing that all of the coordinates of *v* must be 0.

Fix any parameter $q \in \mathcal {Q}_{m,M}^{(n)}$ with $\gamma =0$. We will use the notation $z^c(t):=1-z(t)$ for short.

$$\begin{aligned} \frac{\textrm{d}}{\textrm{d}t}z_{i}^{c}(t)=&-\frac{\textrm{d}}{\textrm{d}t}z_i(t)=-z_i^c(t) \left( \mathcal {A}z(t) \right) _{i} \\ z_{i}^c(t)=&\exp \left( -\int _{0}^{t} \left( \mathcal {A} z(\tau )\right) _{i} \textrm{d}\tau \right) z_{i}^{c}(0) \end{aligned}$$

We give a uniform upper bound on $z_{i}^c(t)$ first. As there is no recovery in the SI model, $z_{i}(t)$ is monotone increasing, so $z_{i}(t) \ge z_{i}(0) \ge m.$ Then

$$\begin{aligned} \left( \mathcal {A}z(t) \right) _i =&\sum _{j \in V} w_{ij} \pi _{j} z_{j}(t) \ge m^2 \sum _{j \in V} \pi _j=m^2, \end{aligned}$$

so

25 $$\begin{aligned} z_{i}^{c}(t) \le&e^{-m^2 t}. \end{aligned}$$

Let $1\!\!1$ denote the all 1 vector. Then

$$\begin{aligned} \int _{0}^{t} \left( \mathcal {A} z({\tau })\right) _{i} \textrm{d}\tau =&\int _{0}^{t} \left( \mathcal {A} 1\!\!1 \right) _i \textrm{d}\tau +\int _{0}^{t} \left( \mathcal {A} z^c({\tau })\right) _{i} \textrm{d}\tau =d_it+O(1), \\ z_{i}^{c}(t)=&\, e^{-d_it+O(1)}z_{i}^{c}(0). \end{aligned}$$

Take a $v \in \mathbb {R}^n$ such that for all $t \in [0,T_0]$ we have $\langle v, z(t) \rangle =0$. From Lemma 2.1, $\langle v, z(t) \rangle =0$ actually holds for any $t \ge 0$.

In particular, since $z(t)=1-z^c(t) \rightarrow 1\!\!1$ as $t \rightarrow \infty$, we must have $\langle v, 1\!\!1 \rangle =0$, which yields $\langle v, z^c(t) \rangle =0.$ Without loss of generality, we assume $d_1<{d_2< } \dots <d_n.$ For $k\in V$ define $\zeta _{k}(t):=\sum _{j \ge k}z_{j}^c(t).$ Due to (25), the slowest exponential decay dominates $\zeta _k(t)$ making

$$\begin{aligned} \lim _{t \rightarrow \infty } \frac{z_{i}^{c}(t)}{\zeta _{k}(t)} =0 \end{aligned}$$

for all $i>k$. Therefore,

$$\begin{aligned} 0=\lim _{t \rightarrow \infty } \frac{1}{\zeta _{1}(t)} \langle {v}, z^c(t) \rangle =\lim _{t \rightarrow \infty } \sum _{i \in V} {v}_{i} \frac{z_{i}^c(t)}{\zeta _1(t)}={v}_1. \end{aligned}$$

Inductively, we may assume ${v}_i=0$ for all $i \le k.$

$$\begin{aligned} 0=\lim _{t \rightarrow \infty } \frac{1}{\zeta _{k+1}(t)} \langle {v}, z^c(t) \rangle =\lim _{t \rightarrow \infty } \sum _{i \ge k+1} {v}_i \frac{z_{i}^c(t)}{\zeta _{k+1}(t)}={v}_{k+1}, \end{aligned}$$

showing that $$v=0$$.

This means *W* is *q*-reconstructible. $\square$

#### Proof of Theorem 3.9

The main idea is the following. We approximate the parameter *p* with a block *homogeneous* $p'$ according to Szemerédi’s regularity lemma. Then we modify it to some $p''$ such that $p''$ is still block *regular* with the same degrees as $p'$, making their epidemic curves identical. In $p'$ each vertex sends the same amount of weight to vertices within the same block (do to homogeneity). In $p''$ on the other hand, we double the weight of half of the edges and make the rest 0 so that the total weight to a block remains the same.

Let $p,p' \in \mathcal {P}_{m,M}^{(n)}$ with corresponding trajectories $z(t), z'(t)$. From Lemma 4.2 and Remark 4.3 we know that for any $\varepsilon >0$ there is a $\delta >0$ such that $\Vert p-p'\Vert _{\square }<\delta$ implies

$$\begin{aligned} \sup _{0 \le t \le T} \Vert z(t)-z'(t) \Vert _{1,\pi }<\frac{\varepsilon }{2}. \end{aligned}$$

From Lemmas 4.2 and 4.4 we know that there exists an $$ n_0$$ such that for any $$n \ge n_0$$ and $$p=(W,q) \in \mathcal {P}_{m,M}^{(n)}$$ there is a block homogeneous $$p'=(W',q') \in \mathcal {P}^{(n)}_{m,M}$$ with $$1 \le K \le K_{\max }(\varepsilon )$$ blocks such that $$\Vert p-p' \Vert _{\square }<\delta .$$

Without loss of generality, we may assume $\delta <\frac{1}{16}m$ and $n_0 \ge 4 K_{\max }(\varepsilon )$. We will modify $W'$ into $W''$ in such a way that $p'':=(\hat{W},q')$ is still block regular with the same partition and $\bar{a}_{kl}$ values, while $W'$ and $\hat{W}$ have a non-vanishing cut distance. This also means that the corresponding trajectory $z''(t)$ is identical to $z'(t).$

Informally, the idea is that every vertex is split into an odd and even version, with weights doubled between vertices of the same parity version, while weights between vertices of different parity versions are set to 0.

Let the size of the block be $|V_k|:=2s_k+r_k$ where $r_{k} \in \{0,1 \}$ and $s_k \in \mathbb {N}.$ We can further divide the blocks into $V_{k,0}, V_{k,1},V_{k,2}$ where $|V_{k,0}|=r_{k}$ and $|V_{k,1}|=|V_{k,2}|=s_k.$

Set $i \in V_{k}, j \in V_l.$ If $i \in V_{k,0}$, then set $\hat{w}_{ij}:=w'_{ij}.$ For $i \in V_{k,1}$,

$$\begin{aligned} \hat{w}_{ij} := {\left\{ \begin{array}{ll} w_{ij}' \text { if } j \in V_{l,0} \\ 2w_{ij}' \text { if } j \in V_{l,1} \\ 0, \textit{ if } j \in V_{l,2} \end{array}\right. } \end{aligned}$$

then for $i \in V_{k,2}$, similarly take

$$\begin{aligned} \hat{w}_{ij} := {\left\{ \begin{array}{ll} w_{ij}' \text { if } j \in V_{l,0} \\ 0 \text { if } j \in V_{l,1} \\ 2w_{ij}' \text { if } j \in V_{l,2}. \end{array}\right. } \end{aligned}$$

Clearly, for any *k*, *l* and $i \in V_k$,

$$\begin{aligned} \sum _{j \in V_l}\hat{w}_{ij} \frac{1}{n}=\sum _{j \in V_l}w'_{ij} \frac{1}{n}{=\sum _{j\in V_l}w_{ij}'\pi _j} \end{aligned}$$

due to $\pi _i=\frac{1}{n}$, so indeed $p''$ is block regular and $z''(t)=z'(t).$ Furthermore, take the sets $S:=\bigcup _{k=1}^{K} V_{k,1}.$ Using (reverse) triangle inequality,

$$\begin{aligned} \Vert \hat{W}-W' \Vert _{\square } \ge&\frac{1}{n^2} \sum _{i,j \in S} \hat{w}_{ij}-w'_{ij}=\frac{1}{n^2} \sum _{i,j \in S} w_{ij}'=\frac{1}{n^2} \sum _{k,l=1}^{K} \bar{w}_{kl}s_{k}s_{l} \\ \ge&\frac{1}{n^2}\sum _{k,l=1}^{K} \bar{w}_{kl} \frac{|V_k|-1}{2} \frac{|V_l|-1}{2} \ge \frac{m}{4n^2} \sum _{k,l=1}^{K} \left( |V_k|-1\right) \left( |V_l| -1\right) \\&=\frac{1}{4}m \frac{n^2-2Kn+K^2}{n^2}\ge \frac{1}{4}m \left( 1-\frac{2K_{\max }(\varepsilon )}{n} \right) \ge \frac{1}{8}m . \end{aligned}$$

From $\Vert W-W' \Vert _{\square } \le \Vert p-p'\Vert _{\square }<\delta <\frac{1}{16}m$ it follows that $\Vert W-\hat{W} \Vert _{\square } \ge \frac{1}{16}m.$

Finally, set $\hat{p}:= (\hat{W},q)$ with trajectory $\hat{z}(t).$ Then

$$\begin{aligned} \Vert p''-\hat{p} \Vert _{\square }=\Vert z(0)-z'(0) \Vert _{1, \pi }+\Vert \gamma -\gamma ' \Vert _{1, \pi } \le d_{\square }(p,p')<\delta , \end{aligned}$$

resulting in

$$\begin{aligned} \sup _{0 \le t \le T} \Vert z(t)-\hat{z}(t) \Vert _{1, \pi } \le&\sup _{0 \le t \le T} \Vert z(t)-z'(t) \Vert _{1, \pi }+\sup _{0 \le t \le T} \Vert z'(t)-z''(t) \Vert _{1, \pi } \\&+\sup _{0 \le t \le T} \Vert z''(t)-\hat{z}(t) \Vert _{1, \pi }< \frac{\varepsilon }{2}+0+\frac{\varepsilon }{2}=\varepsilon . \end{aligned}$$

$$\square $$

### Proofs for Section 3.2

We will prove Theorem 3.12 by showing that the following slightly stronger statement also holds:

#### Theorem 5.1

Assume $0 \le \hat{W}(x,y)=\hat{W}(y,x) \le M$ ($i,j \in V$) a.e. Then for any $\varepsilon >0$ there exists $\delta >0$ such that

26 $$\begin{aligned} \ H_{2,T_0}<\delta \Rightarrow H_{2,T}<\varepsilon \qquad {\forall p \in \mathcal {P}_{0,M}}. \end{aligned}$$

Before proving Theorem 5.1, we make some preparations. Analogous to the Gram matrix (4), define

$$\begin{aligned} G_T(x,y):=&\int _0^T u(t,x)u(t,y) \textrm{d}t, \\ \mathbb {G}_Tf(x):=&\int _{0}^1 G_T(x,y)f(y) \textrm{d}y, \\ \Vert f \Vert _{G_T}^2:=&\langle \mathbb {G}_Tf,f \rangle = \int _0^{T} \langle f, u(t) \rangle ^2 \textrm{d}t. \end{aligned}$$

Clearly $\Vert f \Vert _{G_T}^2 \le T \Vert f \Vert _2^2.$ Due to Lemma 2.1, $\Vert f \Vert _{G_{T_0}}^2=0 \iff \Vert f \Vert _{G_T}^2=0$ $(0\le T_0 \le T).$

Similarly to (10), take $\eta (x)(y):=W(x,y)-\hat{W}(x,y)$ for which we have $\mathbb {W}u(t,x)-\hat{\mathbb {W}} u(t,x)= \langle \eta (x),u(t) \rangle$, and

27 $$\begin{aligned} H_{2,T}^2= \int _{0}^{T} \int _{0}^{1} \langle \eta (x),u(t) \rangle ^2 \textrm{d}x \textrm{d}t=\int _{0}^{1} \Vert \eta (x) \Vert _{G_T}^2 \textrm{d}x. \end{aligned}$$

Throughout the rest of this section, $\rightharpoonup$ denotes weak convergence in $L^2([0,1]).$

#### Lemma 5.2

Assume $f_N \rightharpoonup f$ with $\Vert f_N \Vert _2^2 \le 1$. Then $\Vert f_N \Vert _{G_T}^2 \rightarrow \Vert f\Vert _{G_T}^2$.

#### Proof

$\langle f_N,u(t) \rangle ^2 \le \Vert f_N \Vert _2^2\cdot \Vert u(t) \Vert _2^2 \le 1$, so dominated convergence applies to show

$$\begin{aligned} \lim _{N \rightarrow \infty } \Vert f_N \Vert _{G_T}^2=\int _{0}^{T} \lim _{N \rightarrow \infty } \langle f_N, u(t) \rangle ^2 \textrm{d}t=\int _{0}^T \langle f,u(t) \rangle ^2\textrm{d}t=\Vert f \Vert _{G_T}^2. \end{aligned}$$

$$\square $$

#### Lemma 5.3

Define

$$\begin{aligned} \lambda _N:= \sup _{\Vert f \Vert _2^2 = 1} \frac{\Vert f \Vert _{G_T}^2}{\Vert f \Vert _{G_{T_0}^2}+\frac{1}{N}}. \end{aligned}$$

There exists an $f \in L^2([0,1])$ such that $\Vert f\Vert _2^2 \le 1$ and

$$\begin{aligned} \lambda _N=\frac{\Vert f \Vert _{G_T}^2}{\Vert f \Vert _{G_{T_0}^2}+\frac{1}{N}}. \end{aligned}$$

#### Proof

Clearly $$\lambda _N \le NT.$$

There exists a sequence ${(f_n)_n} \subset L^2([0,1])$ with $\Vert f_n \Vert _2^2= 1$ such that

$$\begin{aligned} \lambda _N=\lim _{n \rightarrow \infty } \frac{\Vert f_n \Vert _{G_T}^2}{\Vert f_n \Vert _{G_{T_0}^2}+\frac{1}{N}}. \end{aligned}$$

Since the unit ball is weakly compact in $L^2([0,1])$, $f_n$ must have a weakly convergent subsequence $f_{n_k} \rightharpoonup f$ with $\Vert f \Vert _2^2 \le 1$, resulting in

$$\begin{aligned} \lambda _N=\lim _{k \rightarrow \infty } \frac{\Vert f_{n_k} \Vert _{G_T}^2}{\Vert f_{n_k} \Vert _{G_{T_0}}^2+\frac{1}{N}} = \frac{\Vert f \Vert _{G_T}^2}{\Vert f \Vert _{G_{T_0}}^2+\frac{1}{N}}. \end{aligned}$$

$$\square $$

Since the value of $p \in \mathcal {P}_{0,M}$ varies, we emphasize that $\lambda _N$ and $G_{T}$ depends on *p*, writing $\lambda _N(p)$ and $G_T(p)$ respectively.

#### Remark 5.4

Based on Remark 4.6:

$$\begin{aligned} \langle f, u(t) \rangle =&\int _{0}^{1}f(x)u(t,x) \textrm{d}x=\int _{0}^{1} f^{\phi }(x) u^{\phi }(t,x) \textrm{d}x=\langle f^{\phi },(u(t))^{\phi } \rangle ,\\ \Vert f \Vert _{G_T(p)}^2=&\int _{0}^{T} \langle f, u(t) \rangle ^2 \textrm{d}t =\int _{0}^{T} \langle f^{\phi },(u(t))^{\phi } \rangle ^2 \textrm{d}t= \left\| f^{\phi } \right\| _{G_T\left( p^{\phi }\right) }^2. \end{aligned}$$

#### Lemma 5.5

Define

$$\begin{aligned} \chi _N:=\sup _{p \in \mathcal {P}_{0,M}} \frac{1}{N} \lambda _N(p). \end{aligned}$$

Then $$\chi _N \rightarrow 0$$ as $$N \rightarrow \infty .$$

#### Proof

Clearly $${0\le } \chi _N \le T.$$

Define $\theta :=\limsup _{N \rightarrow \infty } \chi _N$. Indirectly assume $\theta >0$. Then there must be a subsequence $\chi _{N_K}$ such that

$$\begin{aligned} \chi _{N_k} \ge \frac{1}{2}\theta >0 \quad \forall \, k. \end{aligned}$$

Therefore there must be a $p_{N_k} \in \tilde{\mathcal {P}}_{0,M}$ such that

$$\begin{aligned} \bar{\lambda }_{N_k}:=\frac{1}{N_k}\lambda _{N_k}\left( p_{N_k} \right) =\frac{\Vert f_{N_k} \Vert _{G_T\left( p_{N_k} \right) }^2}{N_k \Vert f_{N_k} \Vert _{G_{T_0}\left( p_{N_k} \right) }^2+1} \ge \frac{1}{4} \theta >0 \end{aligned}$$

for some $$f_{N_k} \in L^2([0,1]).$$

Due to Remark 5.4, we may take an arbitrary $\phi _{N_k} \in \Phi ,$ making

$$\begin{aligned} \bar{\lambda }_{N_k}:=\frac{\left\| f_{N_k}^{\phi _{N_k}} \right\| _{G_T\left( p_{N_k}^{\phi _{N_k}} \right) }^2}{N_k \left\| f_{N_k}^{\phi _{N_k}} \right\| _{G_{T_0}\left( p_{N_k}^{\phi _{N_k}} \right) }^2+1} . \end{aligned}$$

Since $\tilde{\mathcal {P}}_{0,M}$ is compact we can choose a suitable subsequence $(N_k')$ and $\phi _{N_k'} \in \Phi$ such that $p_{N_k'}^{\phi _{N_K'}} \overset{\Vert \cdot \Vert _{\square }}{ \rightarrow }p \in \mathcal {P}_{0,M}$. Furthermore, the unit ball of $L^2([0,1])$ is weakly compact and $\left\| f_{N_k'}^{\phi _{N_k'}} \right\| _2= \left\| f_{N_k'} \right\| _2 \le 1$, thus, for some further subsequence $(N_k'')$ we also have $f_{N_k'}^{\phi _{N_k''}} \rightharpoonup f$ with $\Vert f \Vert _2^2 \le 1.$ Let the solutions of (20) with parameters $p_{N_k'}^{\phi _{N_k''}}, p$ be $u_{N_k''}, u$ respectively. Then $u_{N_k''}(t) \overset{L^1([0,1])}{ \rightarrow }\ u(t)$ uniformly in $t \in [0,T]$ due to Remark 4.3.

Notice

$$\begin{aligned}&\left| \left\| f_{N_k''}^{\phi _{N_k''}} \right\| _{G_{T} \left( p_{N_k''}^{\phi _{N_k''}} \right) }^2-\left\| f_{N_k''}^{\phi _{N_K''}} \right\| _{G_{T} \left( p \right) }^2 \right| \le \\&\int _{0}^{T} \left| \left\langle f_{N_k''}^{\phi _{N_k''}},u_{N_k''}(t) \right\rangle ^2 -\left\langle f_{N_k''}^{\phi _{N_k''}},u(t) \right\rangle ^2 \right| \ \textrm{d}t \le \\&\int _{0}^{T} \left| \left\langle f_{N_k''}^{\phi _{N_k''}},u_{N_k''}(t)-u(t) \right\rangle \right| \cdot \left| \left\langle f_{N_k''}^{\phi _{N_k''}},u_{N_k''}(t)+u(t) \right\rangle \right| \textrm{d}t \le \\&2 \int _{0}^{T} \Vert u_{N_k''}(t)-u(t) \Vert _2 \textrm{d}t \le 2 T \sup _{ 0 \le t \le T} \Vert u_{N_k''}(t)-u(t) \Vert _2 \overset{|u_{N_k''}(t,x)-u(t,x)| \le 1 }{\le } \\&2T \sqrt{\sup _{ 0 \le t \le T} \Vert u_{N_k''}(t)-u(t) \Vert _1 } \rightarrow 0, \end{aligned}$$

which implies

$$\begin{aligned} \lim _{k \rightarrow \infty } \left\| f_{N_k''}^{\phi _{N_{k}''}} \right\| _{G_{T} \left( p_{N_k''} \right) }^2=\lim _{k \rightarrow \infty } \left\| f_{N_k''}^{\phi _{N_k''}} \right\| _{G_{T} \left( p \right) }^2=\Vert f \Vert _{G_T(p)}^2. \end{aligned}$$

Define

$$\begin{aligned} \bar{\lambda }:=\lim _{k \rightarrow \infty } \bar{\lambda }_{N_k''} =\lim _{k \rightarrow \infty } \frac{\left\| f_{N_k''}^{\phi _{N_k''}} \right\| _{G_T\left( p_{N_k''}^{\phi _{N_k''}} \right) }^2}{N_k'' \Vert f_{N_k''}^{\phi _{N_k''}} \Vert _{G_{T_0}\left( p_{N_k''}^{\phi _{N_k''}} \right) }^2+1}=\lim _{k \rightarrow \infty } \frac{\Vert f \Vert _{G_T(p)}^2}{N_k''\Vert f \Vert _{G_{T_0}(p)}^2+1}. \end{aligned}$$

If $\Vert f\Vert _{G_{T_0}{(p)}}^2>0$ then the limit is $\bar{\lambda }=0.$ If $\Vert f\Vert _{G_{T_0}{(p)}}^2=0 \iff \Vert f \Vert _{G_T{(p)}}^{2}=0$, then $\bar{\lambda }=\Vert f \Vert _{G_T{(p)}}^{2}=0.$ This, however, contradicts $\bar{\lambda } \ge \frac{1}{4} \theta >0$. $\square$

Finally we can prove Theorem 5.1 which in turn proves Theorem 3.12.

#### Proof of Theorem 5.1

$\Vert \eta (x) \Vert _2 \le \Vert \eta (x) \Vert _{\infty } \le M$ for almost every $x \in [0,1]$. Furthermore, $H_{2,T}^2$ can be expressed as

$$\begin{aligned} H_{2,T}^2=&\int _{0}^1 \Vert \eta (x) \Vert _{G_T}^2 \textrm{d}x \le \int _{0}^{1} \lambda _N \left( \Vert \eta (x) \Vert _{G_{T_0}^2}+\frac{1}{N}\Vert \eta (x) \Vert _2^2 \right) \textrm{d}x \\ \le&\lambda _{N}\underbrace{\int _{0}^T \Vert \eta (x) \Vert _{G_{T_0}}^2 \textrm{d}x}_{=H_{2,T_0}^2}+M^2 \frac{\lambda _N}{N} \le NTH_{2,T_0}^2+M^2 \chi _N. \end{aligned}$$

Fix $\varepsilon >0$. Based on Lemma 5.5 we can choose a large enough *N* such that $M^2 \chi _N<\frac{\varepsilon ^2}{2}.$ Set $\delta :=\frac{\varepsilon }{\sqrt{2NT}}.$ From this we can say that $H_{2,T_0}<\delta$ implies $H_{2,T}<\varepsilon .$ Note that the choice of $\delta$ only depends on $\varepsilon$ and it is independent of the choice of $p \in \mathcal {P}_{0,M}.$ $\square$

### Proofs for Section 3.3

#### Lemma 5.6

28 $$\begin{aligned} \Vert z(t)-z^*(t)\Vert _{\pi ,2}=O(\Delta ). \end{aligned}$$

#### Proof of Lemma 5.6

$$\begin{aligned}&z(t)-z^*(t)=z(0)-z^*(0)+ \sum _{{1\le } i:t_i\le t} (z(t_i)-z(t_{i-1}))-(z^*(t_i)-z^*(t_{i-1}))+O(\Delta _4), \end{aligned}$$

so

$$\begin{aligned}&\Vert z(t)-z^*(t)\Vert _{\pi ,2}\le \\&\Vert z(0)-z^*(0)\Vert _{\pi ,2}+ \max _{1\le i\le J}\frac{1}{t_i-t_{i-1}}\Vert (z(t_i)-z(t_{i-1}))-(z^*(t_i)-z^*(t_{i-1}))\Vert _{\pi ,2} \\&\times \sum _{{1\le } i:t_i\le t} (t_i-t_{i-1}) + O(\Delta _4) \le \Delta _1+\Delta _3 T_0 + O(\Delta _4)= O(\Delta ). \end{aligned}$$

$$\square $$

#### Proof of Theorem 3.14

We have

$$\begin{aligned} \nonumber&|\alpha _j(t_i)-({\mathcal {A}}z(t_i))_j|= O\Bigg (|z_j(t_i)-z^*_j(t_i)|+|\gamma _j-\gamma _j^*|+\\&\frac{1}{t_{i+1}-t_i}|(z_j(t_i)-z_j(t_{i-1}))-(z_j^*(t_i)-z_j^*(t_{i-1}))|+\\&\left| \frac{1}{t_{i}-t_{i-1}}(z_j(t_i)-z_j(t_{i-1}))-\frac{\textrm{d}}{\textrm{d}t}z_j(t_i)\right| \Bigg )=O(\Delta ), \end{aligned}$$

from which

29 $$\begin{aligned}&\sum _{i=1}^J\sum _{j=1}^n |\alpha _j(t_i)-({\mathcal {A}}z(t_i))_j|^2\pi _j (t_i-t_{i-1})= O(\Delta ^2). \end{aligned}$$

We also have

30 $$\begin{aligned}&\sum _{i=1}^J\sum _{j=1}^n |({\hat{\mathcal {A}}}z(t_i))_j-({\hat{\mathcal {A}}}z^*(t_i))_j|^2\pi _j (t_i-t_{i-1})= O(\Delta ^2) \end{aligned}$$

since $\hat{\mathcal {A}}$ is bounded in $\Vert .\Vert _{\pi ,2}$.

Putting (29) and (30) together, we obtain

31 $$\begin{aligned}&(H_{2,T_0}^*(\hat{W}))^2= \sum _{i=1}^J\sum _{j=1}^n |\alpha _j(t_i)-(\hat{\mathcal {A}}z^*(t_i))_j|^2\pi _j (t_i-t_{i-1})=\nonumber \\&\sum _{i=1}^J\sum _{j=1}^n |({\mathcal {A}}z(t_i))_j-({\hat{\mathcal {A}}}z(t_i))_j|^2\pi _j (t_i-t_{i-1})+ O(\Delta )=\nonumber \\&(H_{2,T_0}(\hat{W}))^2+O(\Delta ). \end{aligned}$$

In the last step, the error of the numerical integration is order $O(\Delta _4)$.

(We also implicitly used $(a+b)^2\le 2(a^2+b^2)$ as a version of the triangle inequality for squares; this affects the constant factor in $O(\cdot )$. Also, the formula $(x+\varepsilon )^2=x^2+2x\varepsilon +\varepsilon ^2$ means the final error in (31) is $O(\Delta )$ instead of $O(\Delta ^2)$.) $\square$
